# The protective role of estrogen and estrogen receptors in cardiovascular disease and the controversial use of estrogen therapy

**DOI:** 10.1186/s13293-017-0152-8

**Published:** 2017-10-24

**Authors:** Andrea Iorga, Christine M. Cunningham, Shayan Moazeni, Gregoire Ruffenach, Soban Umar, Mansoureh Eghbali

**Affiliations:** 1 Department of Anesthesiology, Division of Molecular Medicine, David Geffen School of Medicine at University of California, Los Angeles, BH-160CHS, Los Angeles, CA 90095-7115 USA; 2 Present address: Department of Medicine, Division of Gastroenterology/Liver, Keck School of Medicine of the University of Southern California, Los Angeles, CA 90033 USA

**Keywords:** Estrogen, Estrogen receptor alpha, Estrogen receptor beta, GPR30, Cardiovascular disease, Hormone replacement therapy, Fibrosis, Angiogenesis, Oxidative stress, Vasodilation

## Abstract

Epidemiologic studies have previously suggested that premenopausal females have reduced incidence of cardiovascular disease (CVD) when compared to age-matched males, and the incidence and severity of CVD increases postmenopause. The lower incidence of cardiovascular disease in women during reproductive age is attributed at least in part to estrogen (E2). E2 binds to the traditional E2 receptors (ERs), estrogen receptor alpha (ERα), and estrogen receptor beta (ERβ), as well as the more recently identified G-protein-coupled ER (GPR30), and can exert both genomic and non-genomic actions. This review summarizes the protective role of E2 and its receptors in the cardiovascular system and discusses its underlying mechanisms with an emphasis on oxidative stress, fibrosis, angiogenesis, and vascular function. This review also presents the sexual dimorphic role of ERs in modulating E2 action in cardiovascular disease. The controversies surrounding the clinical use of exogenous E2 as a therapeutic agent for cardiovascular disease in women due to the possible risks of thrombotic events, cancers, and arrhythmia are also discussed. Endogenous local E2 biosynthesis from the conversion of testosterone to E2 via aromatase enzyme offers a novel therapeutic paradigm. Targeting specific ERs in the cardiovascular system may result in novel and possibly safer therapeutic options for cardiovascular protection.

## Background

While cardiovascular disease (CVD) is the leading cause of death among women, epidemiologic studies indicate that females prior to menopause are somewhat protected against the development of CVD when compared to men. Women have reduced incidence of CVD when compared to age-matched males and present with CVD 10 years later than men [1]. In response to aortic stenosis, males have significantly more maladaptive cardiac remodeling than women, and transcriptome characterization reveals that fibrosis and inflammation-related genes and pathways are upregulated in males but not in females [2]. Premenopausal women also withstand ischemia/reperfusion (I/R) injury during open heart surgery better than males [3]. Female protection against CVD is associated with sex hormone levels as the incidence and severity of CVD increases in women postmenopause [3], and the prevalence of coronary artery disease (CAD) is greater in young women who had an oophorectomy compared to those with intact ovaries [4]. Taken together, this data has sparked much investigation into the potential of female sex hormones in conferring cardioprotection. The results of years of research into female sex hormones indicate that the main circulating female hormone, estrogen (E2), is cardioprotective through a plethora of mechanisms that are highlighted throughout this review. While there is abundant data supporting E2 as a cardoprotective agent in experimental models of CVD, E2 replacement therapy in postmenopausal women has been very controversial [5]. Furthermore, while postmenopausal women are at greater risk than premenopausal women of developing CVD, women still have significantly lower incidence of CVD compared to age-matched men well beyond menopause [6]. Discrepancies in the development and prognosis of CVD indicate that the cardioprotection conferred by E2 in females is complex and potentially not the only sex-biasing factor, as other sex-biasing factors such as the sex chromosome composition are also known to contribute to sex biases in CVD [6–8]. The purpose of this review is to summarize the role of E2, E2 receptors (ERs), and aromatase in experimental models of CVD as well as highlight the controversies of hormone replacement therapy in women. We also present the sexual dimorphic role of ERs in modulating E2 action in CVD.

## Estrogen and estrogen receptors

Estradiol, also known as 17beta-estradiol or estrogen (E2), is the most abundant form of circulating estrogens and considered the main female hormone. Two other naturally occurring forms occur in lower abundance (estrone (E1) and estriol (E3)), while a third form (estretrol (E4)) is produced only during pregnancy [9]. E2 is predominantly synthesized and secreted by the ovaries in premenopausal women. Some E2 is also produced in other tissue types including adipose, brain, and bone tissues as well as the vascular endothelium and aortic smooth muscle cells [10]. While gonadal E2 acts largely as an endocrine factor affecting distal tissues, extragonadal production of E2 acts locally as a paracrine or intracrine factor in the tissue where it is synthesized [10]. This extragonadal E2 production plays an important role, as it remains the only source of endogenous E2 production in postmenopausal women and men [10].

**Fig. 1:**
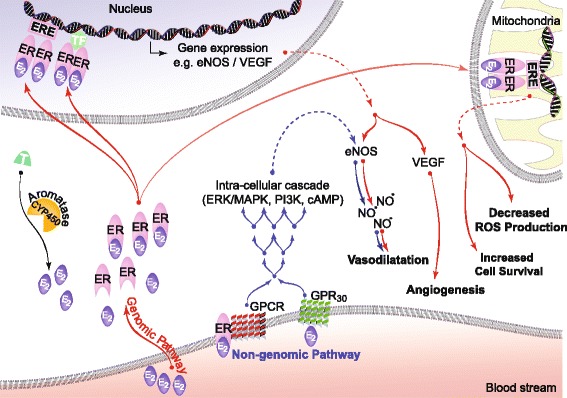
Genomic and non-genomic actions of E2. E2 can regulate gene expression and activity of signaling molecules by binding to ERs via genomic and/or non-genomic pathways. In genomic regulation, binding of E2 to the ER promotes the formation of homo/hetero dimers, translocation to the nucleus and direct binding to estrogen response elements (ERE), or to transcription factors which regulate transcription of its target genes including VEGF, a pro-angiogenic factor. In non-genomic regulation, binding of E2 to ERs and GPR30 at the plasma membrane leads to activation of MAPK/ERK/PI3K/cAMP, which induce gene expression including eNOS, a potent vasodilator. E2 also binds to ERs localized on the mitochondrial membrane improving mitochondrial function by decreasing ROS production and increasing cell survival. Local E2 biosynthesis from the conversion of T to E2 via aromatase (CYP450) is also shown. Genomic pathways are shown in red arrows, whereas non-genomic pathways are shown in blue arrows. Abbreviations: E2 estrogen, ER estrogen receptor, ERE estrogen response element, T testosterone, GPCR G-protein-coupled receptor, PI3K phosphoinositide 3-kinase, MAPK mitogen activated protein kinase, AKT protein kinase B, VEGF vascular endothelial growth factor

E2 binds to the traditional ERs, ERα and ERβ, as well as the newly identified G-protein-coupled ER (GPR30) [11] (Fig. 1). Binding to ERα and ERβ can confer both genomic and rapid non-genomic action [12]. In the genomic pathway, E2 binding triggers intracellular localization of ERα and ERβ, which dimerize and enter the nucleus. Once in the nucleus, they bind to E2 response elements (ERE), or activator protein-1 (Ap1) and specificity protein-1 (Sp1), on the promoter of E2-responsive genes to regulate transcription. E2 also binds to membrane-bound ERα and ERβ receptors as well as GPR30 to rapidly activate nuclear transcription factors via the MAPK pathway. The E2 pathway is summarized in Fig. 1.

While still considered controversial by some, all three E2 receptors have been identified as functional and present in adult cardiomyocytes [11, 13–15]. The role of each ER and the mechanism by which it confers cardioprotection is actively being investigated in various animal models, and the current findings will be summarized in the following sections of this review.

## Estrogen, mitochondria, and oxidative stress

Heart failure (HF) and ischemic heart injury are linked to several bioenergetic abnormalities including decreased energy metabolism, increased apoptosis, production of reactive oxygen species (ROS), and dysfunctional calcium signaling. These abnormalities can largely be attributed to changes in mitochondrial homeostasis [16, 17]. Both traditional ERs and GPR30 are localized on the mitochondrial membrane in cardiac tissue, and activation of these receptors via E2 can trigger transcriptional changes in nuclear and mitochondrial genes influencing mitochondrial function, cell survival, and ultimately cardioprotection [18, 19]. While the beneficial role of E2 in cardioprotection has been demonstrated in both male and female rodents, there is growing evidence demonstrating that E2-induced cardioprotection via mitochondrial homeostasis and oxidative stress may exhibit sex differences.

Lagranha et al. found that adult female rats are protected against ex vivo I/R injury in the presence of E2 as they had smaller infarct size and increased cardiac contractility compared to female rats without gonadal E2 (post ovariectomy, OVX) [20]. Male rats with E2 treatment were also protected against I/R injury with smaller infarct and higher cardiac contractility than non-treated controls. Mitochondria from both females and E2-treated males had increased levels of PKC-dependent phosphorylation of aldehyde dehydrogenase 2 (ALDH2) resulting in increased ALDH activity. Activation of ALDH has previously been reported to protect the heart against ischemic damage [21]. Lagranha et al. also investigated if E2 conferred cardioprotection via activation of PI3K signaling pathway. Pretreatment of hearts with the PI3K inhibitor wortmannin diminished the cardioprotection in female rats following I/R injury as well as reduced p-ALHD2 [20]. This study also linked increased p-ALHD2 to decreased ROS production and found that cardiomyocytes from female rats had less ROS production than cardiomyocytes from male rats following I/R injury [20].

A study by Zhai et al. highlighted the protective role of ERα in male mice following global myocardial I/R by comparing male ERα knockout (ERKO) and wild-type mice [22]. Following ischemia, the hearts of ERKO mice had lower functional recovery, higher incidence of fibrillation and/or tachycardia, and a significantly lower coronary flow rate leading to larger infarct size compared to wild-type mice. Transmission electron microscopy revealed that ERKO hearts had swollen and fragmented mitochondria with loss of matrix and ruptured cristae [22]. Functionally, the mitochondria isolated from ERKO hearts were found to have more severe respiratory dysfunction than wild-type controls [22]. This study demonstrates that ERα plays a cardioprotective role against I/R injury in male mice and that the abolishment of ERα promotes I/R injury via impaired calcium influx and mitochondrial dysfunction. The importance of ERα in mediating E2 cardioprotection has also been identified in females as well. In an in vivo model of I/R using adult female rabbits, acute pretreatment with E2 or ERα-specific agonist PPT, but not ERβ-specific agonist DPN, significantly decreased infarct size suggesting that ERα plays a significant role in the acute cardioprotective action of E2 [12, 23].

E2 also mediates cardioprotection through GPR30 activation on the mitochondrial surface. Bopassa et al. found that during I/R injury, GPR30 activation by E2 in adult male mice results in cardioprotection by inhibiting the opening of the mitochondrial permeability transition pore (mPTP) via activation of the ERK pathway [11]. E2 binding to GPR30 activates adenylyl cyclase, mitogen-activated protein kinases (MAPK), and extracellular signal-regulated kinases, ERK1 and ERK2, resulting in mobilization of intracellular calcium [24]. The mPTP appears to play a pivotal role in apoptosis following I/R [25]. During ischemia, the mPTP remains closed and opens during the first few minutes of reperfusion due to oxidative stress, calcium overload, and ATP depletion [26]. Bopassa et al. found that in the presence of the GPR30 agonist G-1, mouse hearts had better functional recovery as well as a smaller infarct size. Furthermore, the presence of G-1 allowed for the mitochondria to uptake more calcium prior to the opening of the mPTP. All the protective effects of GPR30 activation were blocked by ERK inhibition indicating that GPR30 confers cardioprotection by inhibiting the opening of the mPTP via ERK activation [11].

In agreement with the beneficial role of E2 on the heart, studies have shown a detrimental effect of E2 depletion via OVX on cardioprotection. Pavón et al. found that following OVX, mitochondria from adult female rats have decreased Ca^2+^ retention capacity, which is restored when mitochondria are incubated with E2, thus indicating a protective role of E2 against ischemic injury [27]. A later study by Pavón et al. found that in the absence of E2, female rats also exhibit increased mitochondrial dysfunction: mitochondria were more susceptible to stress-induced dysfunction of complex I and exhibited significant changes in the oxidative phosphorylation-related proteins including cytochrome c oxidase and ATP synthase [28]. Finally, Pavón et al. also reported that E2 is protective against damaging oxidative stress resulting from lipid peroxidation and free radical formation. They showed that levels of thiobarbituric acid reactive substances (TBARS) were increased following I/R injury in OVX females when compared to intact females. The expression of TBARS was also greatly reduced in intact females compared to intact males [27]. A study by Liu et al. suggests that higher levels of circulating E2 in female rodents may protect against cardiovascular stressors due to increased antioxidant expression [30]. Intact female mice were found to have increased cardiac levels of the antioxidant mitochondrial superoxide dismutase 2 (SOD2) when compared to male mice. Following OVX, SOD2 expression decreased to match that of males, and E2 therapy in OVX female mice resulted in increased SOD2 expression. Furthermore, OVX mice fed high-fat diet produced more aortic ROS than intact and E2-treated females fed high-fat diet. E2 stimulated the expression of SOD2 through both classical ERs [30].

A study by Wang et al. further highlighted the antioxidative effects of E2 in cardioprotection. The study found that activation of ERα by E2 in cardiomyocytes decreased levels of microRNA 22 (miR-22) through Sp1 coactivation [29]. Decreased miR-22 expression resulted in an upregulation of cystathionine γ-lyase (CSE) in cardiomyocytes. CSE is known to be upregulated in the myocardium of female rats and in rats treated with E2 and confers cardioprotection by increasing synthesis of the antioxidant hydrogen sulfide [30]. Additionally, female cardiomyocytes are also protected against oxidative stress via ERα-mediated activation of AKT/GSK-3β and AKT/Bcl-2 and inhibition of caspase 3 [31].

**Fig. 2:**
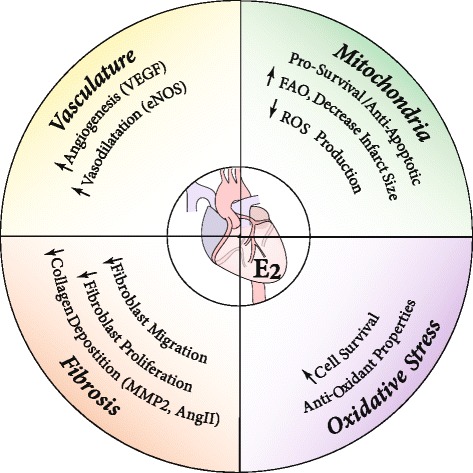
Summary of the likely protective mechanisms of estrogen against cardiovascular disease. Illustration of the possible beneficial effects of estrogen for the treatment of cardiovascular diseases such as ischemic heart disease and heart failure. The protective effect of estrogen in cardiovascular disease is associated with reduced fibrosis, stimulation of angiogenesis, and vasodilation, improved mitochondrial function, and reduced oxidative stress. Abbreviations: VEGF vascular endothelial growth factor, eNOS endothelial nitric oxide synthase, AngII angiotensin II, MMP2 matrix metalloproteinase 2, ROS reactive oxygen species, FAO fatty acid oxidation

In summary, E2 increases cardioprotection against I/R injury and high-fat diet by improving mitochondrial function and reducing ROS mainly through ERα and GPR30 (Fig. 2). E2-enhanced mitochondrial structure and function by activating the cardioprotective signaling pathways (PI3K/ERK1/2) and inhibiting mPTP opening. E2 reduces ROS production and protects against oxidative stress by increasing the production of powerful antioxidants SOD2 and hydrogen sulfide.

## Estrogen and fibrosis

Myocardial extracellular matrix (ECM) proteins, matrix metalloproteinases (MMPs), growth factors, and cytokines are mainly produced by cardiac fibroblasts, all of which contribute to the myocardial structure maintenance as well as cardiac remodeling in diseased hearts [32]. Cardiac remodeling is manifested by changes in the size, shape, and function of the heart including cardiomyocyte hypertrophy, fibroblast proliferation, fibrosis, an accumulation of fibrillar collagens, and myocyte apoptosis [33]. Increased expression of fibrillar collagens leads to stiffening and electrical uncoupling of the cardiac muscle impeding both relaxation and contraction of the heart. Furthermore, increased levels of fibrosis cause a decrease in angiogenesis in the heart as well as an increase of oxygen diffusion distance, thus leading to myocyte hypoxia [34].

The normal ECM, mainly composed of type I and type III collagens, plays an important adaptive role in HF by preventing excessive dilatation when ventricular overload occurs. An imbalance in the synthesis and/or inhibition in the expression of ECM proteins results in fibrosis. In the pressure overloaded ventricles, cardiomyocyte hypertrophy is accompanied by increased collagen deposition between and around myocytes, resulting in interstitial and perivascular fibrosis [35]. The ECM is enzymatically digested by MMPs, and an imbalance of collagen deposition and the activity or expression of MMPs can lead to cardiac remodeling. In the normal heart, the majority of cells are fibroblasts [36], and external stressors cause fibroblasts to change their phenotype to myofibroblasts, which plays a pivotal role in inflammation and fibrosis [32].

A study by Lee and Eghbali-Webb first investigated the expression of the classic ERs in cardiac fibroblasts isolated from female rats. The study found that both ERα and ERβ are expressed in fibroblasts, with the predominant receptor being ERβ which is expressed in the cytosol and nucleus [37]. Lee and Eghbali-Webb also found that treatment of fibroblasts with E2 increases DNA synthesis, which was prevented in the presence of tamoxifen, a selective ER modulator with high inhibitory affinity for both ERs in the heart. This upregulation of DNA synthesis occurs through nuclear translocation of receptor protein and activation of MAPK [37]. These data suggest that E2 acts on the fibroblasts to regulate ECM remodeling and modulate myocardial function via ER and MAPK-dependent mechanisms. Paradoxically, another early study by Dubey et al. found that E2 inhibited fetal calf serum-induced fibroblast proliferation and collagen synthesis in both male and female rats [38]. Furthermore, the E2 metabolites 2-hydroxyestradiol and 2-methoxyestradiol were found to be even more potent than E2 in inhibiting fibroblast proliferation and collagen synthesis, and these effects were enhanced in the presence of progesterone and 4-hydroxytamoxifen (a high-affinity ER ligand) [38]. Considering that the results were similar in both sexes, E2 inhibition of fibroblast proliferation is most likely not acting in a sex-specific manner [38].

Recent studies further support the anti-fibrotic effect of E2 in the heart. Pedram et al. found that ERβ activation prevents cardiac fibrosis by blocking the effects of angiotensin II (AngII) and endothelin-1 (ET-1)-induced pro-fibrotic signaling in female mice [39]. AngII and ET-1 promote cardiac fibrotic deposition by driving the transition of fibroblasts to myofibroblasts and stimulating the synthesis of transforming growth factor-β1 (TGFβ1), a potent inducer of cardiac fibrosis. Upregulation of TGFβ1 in the myofibroblast leads to production of vimentin, fibronectin, and collagens I and III. E2, as well as an ERβ agonist, inhibit TGFβ1 through cAMP and protein kinase A. E2 inhibits fibrosis through ERβ as E2 administration was shown to prevent AngII-induced cardiac fibrosis in female OVX wild-type (WT) mice but not in ERβ knockout mice [39]. Therefore, this study demonstrated the strong anti-fibrotic effects of ERβ activation via E2.

Recent work by our lab found that E2 treatment reverses fibrotic deposition induced by the transaortic constriction model of HF in male mice [40]. E2 treatment diminished the expression of pro-fibrotic genes that are increased in response to cardiac pressure overload including collagens I and III, TGFβ1, lysil oxidase, and fibrosin. E2 treatment resulted in an upregulation of ERβ transcript expression which could elucidate the mechanism by which E2 exerts its effects inhibiting fibrosis [40]. Our study further contributed to the growing body of evidence highlighting E2 as a powerful inhibitor of profibrotic genes that are detrimental to cardiac function.

To study the effect of ERs on fibrosis in the in vivo I/R injury model, mice with overexpression of either ERα or ERβ were subjected to left anterior descending (LAD) coronary artery ligation and subsequent reperfusion. Mahmoodzadeh et al. found that cardiac fibrosis was attenuated in female mice overexpressing ERα after they were subjected to LAD ligation [41]. This suggests that ERα has an important role in protecting the hearts of female rodents against ischemic insults. Another study by Schuster et al. examined the role of ERβ in regulating myocardial infarct size post-LAD [42]. They found that cardiomyocyte-specific ERβ overexpression led to improved heart function and survival in both sexes. Interestingly, male mice with ERβ overexpression had increased left ventricular (LV) volume and EF with reduced cardiac fibrosis when compared to female mice overexpressing ERβ.

The more recently investigated GPR30 may also play a role in conferring cardioprotection via anti-fibrotic mechanisms. Wang et al. found that GPR30 activation via G-1 prevented cardiac fibroblast proliferation and fibrosis both in vitro and in vivo. They demonstrated that cultured cardiac fibroblasts from male rats displayed GPR30 at the cell surface and treatment with G-1 reduced proliferation. In vivo, G-1 treatment attenuated the adverse interstitial collagen deposition and fibroblast proliferation resulting from high-salt diet or E2 depletion from OVX in female rats [43].

MMP-2, which exhibits increased expression and activity following aortic stenosis, myocardial infarction (MI), and LV hypertrophy, is believed to have an adverse effect on cardiac remodeling [44–47]. Furthermore, constitutive expression of MMP-2 causes systolic dysfunction and severe remodeling [48], while its inhibition attenuates cardiac remodeling and improves survival after MI or pressure overload [49–51]. Mahmoodzadeh et al. found that E2 significantly reduced MMP-2 gene expression in male and female adult rat and human fibroblasts via activation of the classic ERs. This effect was due to E2-dependent phosphorylation of the transcription factor Elk-1 via the MAPK signaling pathway [44]. Thus, E2-induced repression of MMP-2 may contribute to sex differences in fibrotic processes, although the authors did not note sex differences in MMP-2 expression.

Lastly, our group has shown that E2 and ERβ activation reverse right ventricular failure caused by pulmonary hypertension (PH) in intact male rats [52]. Right ventricular (RV) failure was associated with upregulation of ECM-interacting cardiac fetal gene osteopontin. We found that the transcript levels of two novel ECM-degrading disintegrin-metalloproteinases (ADAM), ADAM15 and ADAM17, as well as osteopontin and phosphorylated AKT were all elevated in the failing RV and E2 therapy after the onset of PH reversed these effects likely through ERβ activation [52].

In summary, E2 has been demonstrated to have a significant role in ECM remodeling by acting on fibroblasts via ER and MAPK-dependent mechanisms (Fig. 2). Recent data demonstrates that E2 protects against cardiac fibrosis and harmful ECM remodeling by altering fibroblast proliferation and inhibiting various pro-fibrotic genes.

## Estrogen and angiogenesis

By definition, angiogenesis is the physiological process by which new blood vessels form from pre-existing vessels. E2 has previously been shown to be pro-angiogenic in various tissues including the uterus, breast, brain, and limbs [53–55]. Increased cardiac angiogenesis is necessary to maintain LV function during adaptive hypertrophy and compensated heart hypertrophy as the larger LV has an obvious need for more blood vessels [56, 57]. However, an imbalance between cardiac growth and neoangiogenesis over time can induce the progression of compensated heart hypertrophy to HF [58]. It is therefore postulated that stimulation of cardiac neoangiogenesis could be vital in preventing the transition to HF.

Several agents have been shown to induce angiogenesis. The fibroblast growth factor (FGF) family, particularly FGF-1 and FGF-2, are potent angiogenic growth factors that promote endothelial proliferation and organization into tubular structures [59, 60]. Vascular endothelial growth factor (VEGF) is an important angiogenic factor and a critical determinant of capillary growth and density [61]. When bound to its receptor, VEGF starts a signaling cascade which stimulates nitric oxide synthase 3 (eNOS) and nitric oxide (NO) production, endothelial cell proliferation, migration of tube structures, and differentiation into mature blood vessels [61, 62]. We recently showed that VEGF transcript levels are significantly downregulated (~ 5-fold) in male mice with HF and exogenous E2 treatment after establishment of HF was able to restore VEGF expression in the LV [40]. Furthermore, we showed that E2 therapy in male mice significantly enhanced capillary density by ~ 4-fold compared to the HF group alone [40]. Stimulation of cardiac neoangiogenesis by E2 in HF is not only limited to the LV, as E2 also stimulates angiogenesis in RV failure secondary to PH in male rats [67]. Both studies found that E2 was no longer able to rescue HF in the presence of an angiogenesis inhibitor, which indicates that E2 protects against LV and RV HF in male rodents by stimulating angiogenesis [40, 63].

In agreement with our finding, E2 has also been shown to promote vascularization in the experimental model of myocardial ischemic injury. Iwakura et al. showed that E2 augments the incorporation of endothelial progenitor cells (EPCs) in the sites of cardiac ischemic vascularization, resulting in protection from ischemic injury [64]. Using OVX female mice pretreated with placebo or E2 prior to MI induced by LAD ligation, the group found that E2 induced a significant increase in circulating EPCs, which led to an increase in cardiac capillary density. The study also found a greater preponderance of EPCs in the E2-treated animals at the ischemic sites [64]. E2 failed to increase homing of EPCs to the injured myocardium in eNOS-null mice [68]. Therefore, E2 maintains the integrity of ischemic heart tissue by increasing the mobilization and incorporation of EPCs by eNOS. The same group then investigated the role of ERα and ERβ in the E2-induced mobilization of EPCs, which favorably affected neovascularization post cardiac ischemic injury [65]. The study found that the E2-induced endothelial migration, tube formation, and adhesion were impaired when using EPCs derived from ERα or ERβ KO female mice; however, the impairment was more severe when EPCs were derived from ERα KO mice. When bone marrow was transplanted from either ERα or ERβ KO female mice into WT female mice, capillary density at the ischemic border zone was significantly reduced when compared to bone marrow transplanted from WT female mice. ERα transcript levels were more abundant than ERβ in EPCs, and VEGF expression was downregulated only in the ERα knockout EPCs both in vivo and in vitro when compared to WT [65]. Thus, both ERα and ERβ contribute to E2-induced EPC mobilization and preservation of cardiac function in female mice following MI, but ERα plays a more predominant role in this cardioprotective process.

Interestingly, a study by Mahmoodzadeh et al. using intact male and female mice with a cardiomyocyte-specific overexpression of ERα found that ERα increases expression of angiogenesis and lymphangiogenesis markers (such as VEGF and Lyve-1) and neovascularization in the peri-infarct area following MI in a sex-specific manner. Only female mice overexpressing ERα exhibited 100% survival 14 days post-MI concomitant with reduced pathological cardiac remodeling when compared to male mice overexpressing ERα and wild-type mice of both sexes [41]. Thus, ERα overexpression increased cardiac angiogenesis and induced cardioprotection in females, but was not sufficient to confer cardioprotection in males which is likely a result of the increased circulating E2 in females.

In summary, stimulation of neoangiogenesis by E2 therapy in the heart plays an important role in preventing the transition from compensated hypertrophy to HF in both the LV and RV. E2 therapy in HF rodents stimulates neoangiogenesis by enhancing VEGF expression, which is normally suppressed during HF, and increasing capillary density in the heart (Fig. 2). Induction of neoangiogenesis via E2 therapy is dependent on eNOS activation, as eNOS-null mice do not exhibit the pro-angiogenic effects following E2 therapy. Moreover, ERα plays a more dominant role than ERβ in E2-induced EPC activation, upregulation of VEGF transcripts, and preservation of cardiac function post MI, and overexpression of ERα in cardiomyocytes is protective against MI in female but not male mice.

## Estrogen and vasodilation

Under healthy conditions, the vascular endothelium plays an important role in maintaining vascular tone and blood flow. When dysfunctional, the endothelium promotes inflammation, prothrombotic factors, and vasoconstriction, which increase the risk of developing CVD [66–69]. Reports show that E2, when bound to either classical ERs or GPR30, can help maintain endothelial homeostasis and vasodilation by activating the transcription of eNOS and subsequent upregulation of NO through genomic and non-genomic pathways [5, 70–72]. While the effect of E2 on the vascular endothelium is largely time and tissue dependent, studies show that some of the cardioprotection conferred by E2 may be a result of increased vasodilation.

While there is ample evidence supporting NO signaling through ER activation, the mechanisms by which E2 exerts its powerful vasoactive effects are not straightforward. White et al. found that E2 relaxed porcine coronary arteries in an endothelium-independent and dose-dependent fashion; however, when arteries were pretreated with agents to uncouple NO production from eNOS, E2 elicited dose-dependent contractions of arteries [73]. After E2-induced coronary contraction had reached its maximum levels, an inhibitor of superoxide (O_2_^−^, tempol) was able to reverse this response by reducing O_2_^−^. Thus, the authors proposed that acute E2 administration produces both coronary vasodilation and vasoconstriction via NO and O_2_^−^, respectively [73].

In an attempt to investigate the role of ERα and ERβ in NO stimulation and cardioprotection, several groups studied the effect of OVX, E2, and selective ER agonists on vasodilation and hypertension in spontaneously hypertensive rats. Treatment of spontaneously hypertensive OVX female rats with E2 or a selective ERα agonists resulted in improved endothelial function, reduced cardiac hypertrophy, and improved cardiac outcomes when compared to OVX rats without treatment [74, 75]. While Widder et al. found that treatment with E2 or selective ERα agonist resulted in increased eNOS expression in the aorta, there was no statistically significant attenuation of hypertension when compared to OVX rats in this study or in a later study by Pelzer et al. [74, 75]. A follow-up study by Jazbutyte et al. revealed that administration of a selective ERβ agonist to spontaneously hypertensive OVX female rats attenuated hypertrophy, lowered peripheral artery resistance, and decreased systolic blood pressure [76]. Taken together, these studies indicate that E2 improves cardiovascular function in spontaneously hypertensive female rats via both classical ERs, although ERβ elicits a stronger antihypertensive effect. In the AngII-induced cardiac hypertension model, however, Xue et al. found that AngII-induced hypertension was greater in OVX and ERα KO female mice than in intact WT female mice suggesting that E2 acts mainly through ERα to reduce AngII-induced hypertension [77].

In the context of I/R injury, Gabel et al. also concluded that ERβ confers cardioprotection in females by modulating the expression of eNOS and other genes involved in fatty acid metabolism [78]. In this study, gonadally intact males treated with isoproterenol immediately prior to ex vivo ischemia exhibited significantly lower functional recovery following reperfusion than intact females. Transgenic female ERα-KO mice exhibited similar functional recovery to WT females, while female ERβ-KO mice exhibited diminished functional recovery similar to that of WT males. Notably, cardiac eNOS protein expression was found to be significantly lower in the ERβ-KO mice when compared to female WT and ERα-KO mice, indicating that the ERβ-mediated expression of eNOS may be responsible for sex differences in functional recovery following I/R injury [78]. In a separate study, female ERβ-KO mice were also found to exhibit abnormal vascular function, hypertension, increased mortality, and aggravated heart failure following MI when compared to female WT controls [79]. Interestingly, Pelzer et al. did not find significant differences in LV eNOS expression between groups [79].

Similarly, Nikolic et al. found that 2-week pretreatment with DPN, an ERβ agonist, is cardioprotective in I/R injury in OVX female mice [79]. Gene profiling in this experimental model revealed that treatment with DPN is associated with a significant increase in cardioprotective genes, including those encoding NO biosynthesis and anti-apoptotic proteins [80]. Lin et al. showed that chronic E2 or DPN treatment also leads to activation of protein S-nitrosylation and cardioprotection, which was blocked by eNOS inhibition [81]. Taken together, these data indicate that chronic E2 exposure protects the heart largely via ERβ activation and subsequent NO signaling.

Studies suggest that classical ERs are not solely responsible for E2-induced vasodilation and that GPR30 activation can also impact vascular tone. Lindsey et al. found that activation of GPR30 via G-1 induced similar vasodilatory effects as E2 in isolated aortic rings from both intact and OVX female rats [82]. Pretreatment with GPR30 antagonist, G15, successfully blocked G-1- and E2-induced vasodilation. Similarly, removal of the endothelium or pretreatment with l-NAME, a constitutive eNOS inhibitor, partially attenuated vasorelaxation. These data suggest that GPR30 activation by E2 or selective agonist induces vasorelaxation in female rodent aortas through a partially endothelium and eNOS-dependent mechanism [82].

More recently, Fredette et al. elucidated the mechanism by which E2 and G-1 induce vasodilation in male human endothelial cells and GPR30-KO mice [83]. The study demonstrated that GPR30 stimulates endothelial NO production via activation of the c-Src/EGFR/PI3K/ERK1/2 signaling pathway. E2 and G-1 treatment induced NO release in cultured endothelial cells and extracted aortic rings from WT mice. In GPR30-KO mice, the vasodilatory effect of G-1 was completely abrogated while the effect of E2 was reduced by 50% [83].

The significant involvement of GPR30 in eliciting a vasodilatory response may contribute to some of the sex differences in CVD. A study by Lenhart et al. reported that female mice have more membrane-localized GPR30 when compared to male mice [84]. Furthermore, they found that GPR30 directly interacts with receptor active modifying protein 3 (RAMP3), a protein known to affect the trafficking and activity of several GPCRs. After subjecting male and female WT and Ramp3-KO mice to GPR30 activation via G-1, the authors found the cardioprotection conferred by G-1 to be both Ramp3 and sex specific [84].

Sudhir et al. found that E2 induced a significant increase in coronary cross-sectional area, flow velocity, and volumetric blood flow in male and female canine coronary arteries that was not attenuated by pretreatment with l-NAME or the classic ER antagonist, ICI 182,780 [85]. The investigators thus conclude that acute E2-induced dilation of coronary arteries is endothelium-independent and is not mediated via the classic intracellular E2 receptors, but presumably via non-genomic mechanisms involving GPRs at the plasma membrane [85].

In addition to stimulating eNOS production, E2 has also been shown to enhance vasodilation through inhibition of the angiotensin pathway. Isolated RNA from different tissues (kidney cortex, kidney medulla, lung, and aorta) of OVX female rats treated with E2 for 21 days revealed a downregulation of angiotensin-converting enzyme (ACE) transcript levels [86]. Thus, downregulation of ACE with a consequent reduction in the circulating levels of the vasoconstrictor AngII could be another mechanism by which E2 confers its beneficial cardiovascular effects.

In summary, studies suggest that E2 plays a very dynamic role in modulating vasorelaxation, vasoconstriction, and endothelial function via largely eNOS-dependent mechanisms (Fig. 2). This mechanism may contribute to the cardioprotective effect of E2 as E2 reduced hypertrophy and increased cardiac function in the spontaneous hypertensive rat model and in models of I/R injury. Further studies using animal models of hypertension found that stimulation of classical ERs may also attenuate hypertension. Stimulation of GPR30 by E2 or GPR30-specific agonists also stimulated eNOS and NO production in a sex-specific manner. By reducing the expression of ACE, E2 treatment also results in lower levels of AngII and reduced vasoconstriction.

## Aromatase and the cardiovascular system

While the main site of E2 production is the female ovary, peripheral tissues can also act as sites of local E2 production by converting androgens into E2 via the aromatase enzyme [87]. Aromatase is widely distributed in gonadal and extragonadal tissues including the bone, brain, adipose tissue, and blood vessels [88]. Local production of E2 in these tissues by aromatase plays an important role in the physiological functions of these tissues [89]. For example, the presence of aromatase in the coronary endothelium suggests that the local cardiac conversion of androgens to E2 likely affects cardiac function and structural modeling [90].

Aromatase is encoded by the CYP19 gene, and genetic variations in the CYP19 gene result in alterations of circulating levels of sex hormones [91–93]. Ma et al. suggests that genetic variations in CYP19 might contribute to variations in the pathophysiology of E2-dependent diseases [94]. Wang et al. identified a single nucleotide polymorphism in the CYP19 gene to be associated with a decreased risk of coronary heart disease among a Chinese population in which both sexes were equally represented [95].

Aromatase activity has been confirmed in female rat arterial smooth muscle cells (SMCs) and in bovine coronary endothelial cells (ECs) in vitro [96, 97]. Harada et al. further demonstrated aromatase activity in human male and female arterial SMCs, but not in female ECs, which could potentially indicate autocrine/paracrine E2 activity [89]. It is speculated that E2 produced in vascular SMCs acts to promote cardiac contractility, vasodilation, and collagen synthesis, while also stimulating EC functions such as NO production and angiogenesis [89]. Within vasculature, E2 protects against atherosclerotic progression in experimental animal models of atherosclerosis in both males and females, and aromatase has been shown to play a pivotal role in promoting this vascular protection [98–100]. The ablation of aromatase has also been demonstrated to be associated with increased adiposity [101] and progressive insulin resistance [102], which are significant factors in the development of cardiovascular diseases including atherosclerosis and diabetic cardiomyopathy.

While E2 is largely considered to be protective in both male and female rodents in the context of I/R injury and HF, the role of aromatase in conferring cardioprotection is unclear. Bell et al. investigated the role of aromatase deficiency in I/R injury using aromatase knockout (ArKO) female mice and found that the recovery of left ventricular developed pressure as well calcium handling was substantially improved in ArKO versus wild-type (WT) mouse hearts [103]. Later on, the same group demonstrated that isolated cardiomyocytes from ArKO female mice exhibited greater basal calcium transient amplitude and shortening than in WT [104]. Isolated cardiomyocytes from ArKO female mice exposed to a high-calcium load also showed increased calcium transient and contractile amplitudes. The study concluded that the relative withdrawal of E2 in favor of testosterone may be positive inotropic via optimized calcium handling in response to stress and aromatase inhibition which could lead to cardioprotection [104]. Bell et al. also studied whether upregulation of tissue aromatase expression could improve ischemic resilience in male hearts by subjecting male mice in which aromatase was transgenically upregulated (AROM+) to I/R injury [105]. As expected, male AROM+ mice exhibited markedly lower testosterone and higher E2 levels when compared to wild-type male mice. AROM+ male hearts had better functional recovery post ischemia, whereas female AROM+ hearts did not exhibit the same improvement in post-ischemic function [105]. Thus, these findings demonstrate that aromatase modulates cardiac function in a sex-specific manner [105].

A recent study by our group found that cardiac aromatase transcript levels are reduced in male mice in the TAC-induced model of LV HF [40]. Interestingly, these mice also exhibited decreased local cardiac E2 levels, but not circulating plasma E2. E2 therapy for 10 days after the onset of HF restored aromatase transcript levels in the heart similar to control levels and upregulated local cardiac E2 concentrations. We stipulated that the normalization of cardiac aromatase by E2 could either be attributed to improvement in cardiac function or increased E2 bioavailability in the heart [40]. As the aromatase gene has several half E2 response elements as well as Sp1 and Ap1 elements, it is possible that increased heart E2 concentrations reported in the study post-E2 therapy could have stimulated the transcription of aromatase.

Recent studies have also implicated the role of aromatase in regulating RV function in the Sugen hypoxia rat model, an experimental model of PH that is associated with an elevated RV systolic pressure and RV hypertrophy. Aromatase inhibition using anastrozole (an aromatase inhibitor) reversed adverse RV remodeling in female Sugen hypoxia rats [106]. Studies have also shown that metformin (a drug used to treat type 2 diabetes by controlling blood glucose levels) reversed elevated RV systolic pressure and hypertrophy in Sugen hypoxia female rats, independent of glycemia, without any effects on systemic blood pressure, cardiac output, or heart rate [107]. Interestingly, metformin was shown to inhibit aromatase without any adverse effects on the RV [108]. Taken together, these studies suggest that aromatase inhibition may reverse PH and that metformin could be used to treat PH by inhibiting aromatase activity.

It is well-known that the incidence of cardiovascular events is significantly lower in women when compared to age-matched men even following menopause [109]. In postmenopausal women, peripheral aromatase activity becomes the primary source of E2 synthesis, and studies indicate that aromatase activity may also increase with age. Plasma concentrations of cytokines known to stimulate aromatase activity were shown to increase over time in males and females [87]. Differences in aromatase activity between males and females may contribute to sex biases even after menopause. Aromatase inhibitors, which are used clinically for treatment of breast cancer, have been shown to carry the potential risk of increased cardiovascular events. One clinical study found that treatment with aromatase inhibitors is associated with a higher risk of ischemic heart disease versus tamoxifen, a broad ER antagonist [110]. Another population-based observational study of a heterogeneous population of 74 women with early breast cancer who received adjuvant hormonal therapy and subsequently underwent cardiac angiography concluded that aromatase inhibitors significantly increased the hazard for CAD compared to tamoxifen [111]. These studies, however, are in direct conflict with recent genome-wide association studies that attribute this finding to the cardioprotective effects of tamoxifen versus the detrimental effects of aromatase inhibitors [112].

Interestingly, the concurrent use of aromatase inhibitors and adjuvant radiotherapy for early-stage left-sided breast cancer, in the absence of tamoxifen treatment, yielded a significant decrease in RV systolic function and LV diastolic function when compared to radiotherapy alone [113]. While more long-term studies are necessary, these findings indicate that aromatase inhibition may lead to reduced cardioprotection in females.

Aromatase inhibition may increase the risk of CVD in men as well. A recent intervention pilot study by De Smet et al. investigated the effect of aromatase inhibition on the cardiovascular system on young healthy men 20–40 years old [114]. The study employed 20 participants who were randomized to the aromatase inhibitor group (letrozole, 2.5 mg daily for 7 days) or letrozole plus an E2 patch (75 μg/day for 7 days). The letrozole plus E2 group, which yielded a similar sex steroid profile to that observed in obese men, demonstrated a statistically significant decrease in circumferential strain indicating subclinically impaired function of the LV subendocardial layers [114].

In summary, the aromatase enzyme converts androgens to E2 in many extragonadal tissues. While the bulk of experiments suggest that E2 is cardioprotective, the effect of increased aromatase activity remains controversial. In vasculature, aromatase activity seems to protect against the development of atherosclerosis, and aromatase null mice exhibit increased adiposity and insulin resistance which are risk factors in the development of CVD. Conversely, aromatase null mice also exhibit increased calcium handling and better functional recovery when subjected to I/R injury. In direct contradiction to this finding, aromatase overexpression resulted in better functional recovery following I/R in male mice but not in female mice. Interestingly, aromatase expression is downregulated in HF, yet inhibition of aromatase activity helps to reverse RV HF and hypertrophy in rodents. In humans, the use of aromatase inhibitor in women and men may increase the risk of developing CVD or CVD risk factors. These findings, while contradictory, demonstrate that aromatase may play a role in mediating cardioprotection; however, more research is needed to investigate the nature of aromatase role.

## Controversies regarding the use of estrogen for therapeutic purposes

While the use of E2 therapy in CVD has shown predominantly beneficial effects in animal models, the use of E2 in humans in the form of hormone replacement therapy has been very controversial. The following are the main controversial aspects of hormonal therapy in humans.

### Efficacy of hormone replacement therapy

Two large-scale randomized, double-blind, placebo-controlled clinical trials, the Women’s Health Initiative (WHI) and Heart and Estrogen/progestin Replacement Study (HERS), were designed to examine the efficacy of hormone replacement therapy (HRT) in reducing the risk of ischemic heart disease in postmenopausal women [115]. In WHI trails, postmenopausal women (50–79 years old) without a history of CVD were administered a daily dose of conjugated equine E2 (CEE, 0.625 mg) plus medroxyprogesterone acetate (MPA, 2.5 mg). Unfortunately, the WHI trail was discontinued prematurely due to adverse cardiac events and increased risk of breast cancer [116]. The HERS trial, conducted in postmenopausal women with pre-existing CVD (44–79 years old), found that daily use of CEE (0.625 mg) plus MPA (2.5 mg) had no effect on primary or secondary cardiovascular outcomes [117, 118].

The failure of both trials to support the protective role of HRT in reducing CVD could, in part, be due to the HRT being administered long after the onset of menopause in most of the women enrolled. A new hypothesis deemed the “Critical Window of Hormone Therapy” has recently received a lot of attention. The hypothesis supports the view that HRT could be effective if started early at the onset of menopause [119]. A more recent randomized study that administered HRT to women (42–58 years old) within 6–36 months following the onset of menopause supports the “Critical Window” hypothesis as certain risk factors for CVD were reduced [120]. Another randomized study in women (45–58 years old) also showed a significantly reduced risk of mortality, heart failure, or myocardial infarction if HRT was initiated early after menopause [121].

Another potential reason for the failure of the WHI and HERS trials could be that the hormone dosage and the combination of E2 and progestin employed in WHI and HERS may not have been optimal for preventing or reducing CVD risk. CEE administered alone at a lower dose (0.3 mg/day) was shown to decrease major coronary events in women [122] and coronary artery atherosclerosis in adult female monkeys [123]. Progestins, however, have been shown to downregulate ERs and stimulate direct progestin receptor-mediated effects that oppose estrogenic action [116]. MPA, a progestin administered in conjunction with E2 in the WHI and HERS trial, may also negate any cardioprotective effects of E2 therapy. A randomized placebo-controlled cross-over study showed, however, that oral administration of E2 for 3 months without progestin does not improve coronary flow reserve in postmenopausal women [124].

Currently, the Kronos Early Estrogen Prevention Study (KEEPS) is an ongoing trial testing the effect of HRT in atherosclerosis and coronary calcification in women within 3 years of menopause. KEEPS will study the effect of HRT with or without progestin as well as test different modes of E2 administration (CEE or transdermal E2) [125].

Finally, disparity between the efficacy of HRT in animal studies and the WHI and HERS clinical trials could be due to the downregulation of ERs in aged women. Li et al. found that the expression of ERβ and GPR30, but not ERα, are significantly decreased in the arteries of female rats [126]. Since most effects of estrogen are mediated through its receptors, downregulation of ERs could silence the beneficial effects of E2. Further studies are needed to examine the expression of ERs in aged women and identify if the efficacy of HRT changes with age.

In summary, despite numerous animal studies demonstrating the beneficial cardioprotective effects of E2, the WHI and HERS clinical trials both failed to support the effectiveness of HRT in reducing CVD. However, this may be attributed to several factors including the initiation of HRT long after the start of menopause, the dose, and combination of E2 and progestin. The ongoing large randomized KEEPS trial, which administers E2 with and without progestin early after the onset of menopause, has the potential to shed valuable light on the efficacy of HRT in reducing CVD in early postmenopausal women.

### Arrhythmias, sudden cardiac death, and estrogen

Sex differences exist in the underlying electrophysiology of the heart that may influence the risk of developing arrhythmia and sudden cardiac death. It is likely that these differences are modulated by sex hormones and that sex hormones also affect the presentation of these arrhythmias and the response to antiarrhythmic drugs and therapies [127]. In fact, women have a higher risk of acquired long QT syndrome from antiarrhythmic drugs than men. Thus, understanding the effects of hormones in mediating arrhythmic risk and response to treatment is important in order to provide effective and individualized treatment for patients.

In experimental models of CVD, the role of E2 in reducing ventricular arrhythmias has been controversial. In support of antiarrhythmic effects of E2, Philp et al. examined the effect of E2 on ischemia-induced cardiac arrhythmias and found that administration of E2 before coronary artery ligation exhibited antiarrhythmic activity in a dose-dependent manner in female rats [128]. Interestingly, the antiarrhythmic activity of E2 in the myocardial ischemia setting was greater in female rats than in male rats [128], which could be due to the effects of E2 on the expression and function of ion channels that control cardiac cell excitation and repolarization [129]. Sykes et al. showed that inhibition of E2 synthesis with an aromatase inhibitor triggered cardiac arrhythmias in the developing zebrafish [130]. While these data suggest that E2 is antiarrhythmic, several studies demonstrate that E2 actually promotes ventricular arrhythmias. For example, female rats treated with E2 and subjected to ex vivo ischemia experienced an increase in sustained ventricular arrhythmia upon reperfusion [131]. The pro-arrhythmic action of E2 was due to alterations of calcium handling in cardiomyocytes mediated through ERβ signaling [131]. Interestingly, while E2 increased the incidence and duration of ventricular arrhythmia, the infarct size post-I/R was significantly reduced which highlights the cardioprotective role of E2 in I/R injury [131]. In an in vivo model of drug-induced arrhythmia, E2 was also found to trigger ventricular arrhythmias in both male and female rabbits [132]. Odening et al. showed that E2 also increases ventricular arrhythmias and sudden cardiac death in OVX transgenic long QT rabbits [133].

In the Oregon Sudden Unexpected Death Study (catchment population approximately one million), Narayanan et al. compared cases of sudden cardiac arrest (SCA) with matched controls. A multivariate analysis on testosterone and E2 levels measured from blood samples drawn at the time of the SCA event revealed that higher testosterone levels only in men were associated with lower SCA events. On the other hand, higher estradiol levels in both women and men were associated with higher SCA events [134].

In summary, in the experimental settings of CVD, the role of E2 in ventricular arrhythmias is controversial as E2 was found to be both pro- and antiarrhythmic in females or both sexes depending on the study. In humans, increased plasma E2 levels are found to be associated with higher SCA events in both men and women. Since females are more prone to drug-induced arrhythmia than males [135], further studies are needed to examine whether the female heart has a unique sensitivity to E2 in the presence of certain drugs.

### Estrogen and thrombosis risks

The use of E2 as a therapeutic agent for cardiovascular protection is controversial as the use of oral contraceptives containing E2 has been linked to an increased risk of venous thrombosis which can lead to MI, stroke, and peripheral artery disease. While the intent of the WHI clinical trials was to investigate the role of E2 in postmenopausal women, the investigators did find that the estrogen component of HRT was also associated with increased risk of venous thrombosis. Canonico et al. reported a similar finding [136]. These studies suggest that E2 may have prothrombotic ability, and while controversial and not fully understood, E2 may achieve this by increasing procoagulant factors (factors VII, X, XII, and XIII) and decreasing anticoagulant factors (protein S and antithrombin) [137]. Thus, E2 supplementation via oral contraceptives or HRT enhances the risk of venous thrombosis especially in women with pre-existing coagulation abnormalities. Progestin also contributes to this risk as oral contraceptives or HRT containing third-generation progestogens (desogestrel or gestodene) are associated with a higher risk of venous thrombosis than those containing second-generation progestogens (such as levonorgestrel) [138].

A study by Nayak et al. found that tamoxifen, a well-established cancer drug that reduces the effects of E2, has an antithrombotic effect in mice and human blood samples. In this study, tamoxifen significantly inhibited platelet functions in human plasma while it prolonged tail bleeding time and prevented thrombus formation at injured arterial walls in mice [139]. This study further demonstrates that ER modulation impacts coagulation.

While most evidence suggests that E2 has procoagulatory and prothrombolytic effects, a clinical trial assessing HRT on cardiovascular events in recently postmenopausal women over 10 years did not reveal an increased risk of thrombolytic events. Women receiving HRT early after menopause had a significantly reduced risk of CVD, without any apparent increase in risk of cancer, venous thromboembolism, or stroke [121].

In summary, E2 treatment in the form of oral contraceptives and HRT have been linked to increased risk of venous thrombosis, possibly due to E2's modulatory role on  procoagulant and anticoagulant factors. Research demonstrating that tamoxifen exhibits antithrombotic effects further suggests that ER modulation is involved in coagulation. A long-term clinical trial studying HRT in recently menopausal women, however, was not associated with increased risk of thrombolytic events.

## Conclusions

The protection against cardiovascular disease in women during reproductive age is believed to be related at least in part to E2 since endogenous levels of E2 and the expression of ERs differ considerably between sexes. E2 mediates its cardioprotective actions by increasing angiogenesis and vasodilation and decreasing ROS, oxidative stress, and fibrosis. Through these mechanisms, E2 limits cardiac remodeling and attenuates heart hypertrophy (Fig. 2). Although the use of E2 as a therapeutic agent in humans has remained controversial, targeting specific ERs in the cardiovascular system may result in novel and possibly safer therapeutic options for the use of E2 for cardiovascular protection.

## Abbreviations

ACE: Angiotensin-converting enzyme
ADAM: Disintegrin-metalloproteinase
ALDH2: Aldehyde dehydrogenase 2
AngII: Angiotensin II
Ap1: Activator protein-1
ArKO: Aromatase knockout
AROM+: Mice in which aromatase was transgenically upregulated
CAD: Coronary artery disease
CEE: Conjugated equine estrogens
CSE: Cystathionine γ-lyase
CVD: Cardiovascular disease
E2: 17Beta-estradiol, estrogen
EC: Endothelial cells
ECM: Extracellular matrix
eNOS: Nitric oxide synthase 3
EPC: Endothelial progenitor cells
ER: Estrogen receptors
ERKO: Estrogen receptor α knockout
ERα: Estrogen receptor α
ERβ: Estrogen receptor β
ET-1: Endothelin-1
FGF: Fibroblast growth factor
GPR30: G-protein-coupled estrogen receptor
HERS: Heart and Estrogen/progestin Replacement Study
HF: Heart failure
HRT: Hormone replacement therapy
I/R: Ischemia/reperfusion
KEEPS: Kronos Early Estrogen Prevention Study
LAD: Left anterior descending
LV: left ventricular
MI: Myocardial infarction
miR-22: MicroRNA 22
MMP: matrix metalloproteinaises
MPA: Medroxyprogesterone acetate
mPTP: Mitochondrial permeability transition pore
NO: Nitric oxide
OVX: Ovariectomy
PH: Pulmonary hypertension
RAMP3: Receptor active modifying protein 3
ROS: Reactive oxygen species
RV: Right ventricular
SCA: Sudden cardiac arrest
SMC: Smooth muscle cells
SOD2: Superoxide dismutase 2
Sp1: Specificity protein-1
O_2_^−^: Superoxide
TBARS: Thiobarbituric acid reactive substances
TGF: Transforming growth factor-β1
TGFβ: Vascular endothelial growth factor
VEGFR-2: VEGF binding to its receptor-2
WT: Wild type
WHI: Women’s Health Initiative
